# Reproductive biology in cassava: stigma receptivity and pollen tube growth

**DOI:** 10.1080/19420889.2019.1631110

**Published:** 2019-06-27

**Authors:** L.N. Ramos Abril, L.M. Pineda, I. Wasek, M. Wedzony, H. Ceballos

**Affiliations:** a Cassava Program, International Center for Tropical Agriculture (CIAT), Cali, Colombia; b Universidad Nacional de Colombia, Palmira, Colombia; c Pedagogical University, Cracow, Poland

**Keywords:** Cassava sexual reproduction, controlled pollinations, doubled haploids, embryo sac, nucellar beak, cyathia

## Abstract

Knowledge on the reproductive biology of cassava, relevant to breeders and molecular geneticists, is still limited. Therefore, different studies were carried out to determine the duration of stigma receptivity and the rate of pollen tube growth. Inflorescences were covered for up to 3 days after the first opening of the bracts (e.g. anthesis day) to prevent open pollination. Results indicate that fruit and seed set are drastically reduced when flowers were covered for 2 or 3 days. However, fruits and seeds were obtained even from flowers that had been covered for 3 days after anthesis, although at low frequency. The rate of pollen tube growth was assessed in many combinations of female and male progenitors crossed through controlled pollinations and collecting the pistils at varying hours after pollination (HAP). Pollen tube growth is fast during the first 6 HAP reaching the tip of the nucellar beak. The growth slows down thereafter, taking 10 additional hours to reach the end of the beak. The growth of pollen tubes slows down even further until they enter the embryo sac. Only 10% of samples showed pollen tubes entering the embryo sac between 48 and 66 HAP. Although several tubes may reach the nucellar beak, only one was observed entering the embryo sac. Results, across the different experiments, were highly variable suggesting that the timeline of fertilization is influenced both by genotypic and environmental factors as well as the manual manipulation of inflorescences and cyathia.

## 1. Introduction

Cassava (*Manihot esculenta* Crantz) is a crop of neotropical origin and of significant economic relevance. It is a fundamental food security crop in many regions of the world, particularly Sub-Saharan Africa. Being the second most important source of starch worldwide [1], it is also an important industrial crop. Manihot species grow naturally from the south-western North America to the northern Argentina [2–4]. The last taxonomic revision was performed by Rogers and Appan in 1973. Few changes have been proposed thereafter [5–7].

Cassava can be propagated either vegetatively from stems or from seed, but the former is the most common practice for the commercial production. At harvest, farmers cut young branches and discard them. Before harvesting the roots, the main stems (0.5–2.0 m in length, depending on the cultivar and growing conditions) are cut and tied together in bundles of approximately 50 stems [8]. Sexual reproduction, the key requirement for conventional breeding, is common and relatively easy to achieve [9,10]. Several breeding programs are currently active in generating new genetic variation through crossing [11].

Cassava is a diclinous and monoecious species: either female (pistillate) or male (staminate) flowers are produced in inflorescences (racemes or panicles) within the same plant. The evolution of inflorescence and flower structures in Euphorbiaceae is remarkable when compared with other dicots, and involves numerous organ reductions. Cassava flowers are in fact apetalous. Female flowers are reduced to a pistil that is protected by petal-like bracts of leaf origin. What is commonly identified by breeders and farmers as a male flower is actually an inflorescence of 10 single-stamen flowers. These inflorescences, known as cyathia are also protected by bracts. Male and female cyathia will be treated in this article as if they were single flowers as the distinction is only relevant from the botanical point of view [12].

Synchronization of flowering for planned crosses can be challenging because some clones flower relatively early at 4 or 5 months after planting (MAP), whereas others flower only after 10 MAP. The scarcity of flowers in erect, non-branching types complicates crossing even further. In addition, the time required for the seed to mature is two to three months (depending on environmental conditions and genotype) so it takes generally more than a year to obtain seeds from a planned cross. Several publications illustrate the procedures for controlled pollinations in cassava [9,13–15]. There is no evidence of incompatibility among genotypes. Crosses can be done easily, except for the scarcity or absence of flowers in certain genotypes. There is no evidence of self-incompatibility either, so it is technically possible to make self-pollinations and obtain viable botanical seed [16,17].

Important advances have been made on the reproductive biology of cassava in recent years [18,19]. Nonetheless, there are still important features of the sexual reproduction in cassava that remain unclear. Is the bracts opening of the same functional meaning as the opening of petals and sepals in other species? Female cassava flowers are regarded fertile on the first day of bracts opening and therefore pollinated at noon or afternoon of that day (day of anthesis). However, the fertility of cassava female flowers has never been studied in detail. Similarly, information of pollen tubes (PTs) growth in the pistil is limited for cassava. A clear understanding of the pollination and fertilization processes is critical for developing protocols for the production of doubled haploids through wide crosses or the irradiation of pollen grains [20–22]. Pollination and PT elongation have multiple effects on the pistil physiology [23–25]. In many species, signals elicited by the PT growth are necessary to properly prepare embryo sac for fertilization, sometimes it is even a prerequisite for its full maturation [24,26,27].

The primary objectives of the present study were: **i)** to assess a period of time, in relation to bracts opening (anthesis), in which stigmas remain receptive; **ii)** to study the success of pollination measured as the rate of pollen germination and growth of PT towards the pseudomicropyle; and **iii)** to define the time required for PT to reach the embryo sac in the pistils.

## 2. Material and methods

### 2.1 Plant material

The complete list of clones used in the crosses presented in this publication can be found in Table 1. Clones HMC1, SM1219–9, MNga11 and MCol1505 were selected because they undergo 3–4 flowering events in a growing cycle of one year. In years 2011–2017 these clones were planted every other week and used throughout this study. SM1219-9 is the result of a polycross (open pollination) made in 1988, for which only its female progenitor (CG 1450–4), derived from the Colombian landraces MCol 1505 and MCol 1940, is known. HMC1 is a commercial variety released in Colombia from a cross made in 1980. It was obtained by irradiation of the landrace MCol 1438. MCol 1505 is a commercial landrace of unknown genetic origin. MNga11 is the code name for the accession in the germplasm collection at CIAT of the African genotype 60,444. This clone is known as the model genotype for early and current work on genetic transformation. The remaining genotypes were part of ordinary crossing nurseries of the cassava breeding program and were used because they happened to be flowering when experiments took place (Table 1).

**Table 1.** Material used in experiments and the experiments sizes. For experiments 4 and 5, the number of pollinations made with different male progenitors is specified within parenthesis.

Female parent	Male parent	Number of flowers	Trait assessed
**Experiment 1**			
MNga 11	Trial 1a: open pollination	483	Number of fruits
SM1219–9		438	
Mixture 1^a^		417	
MNga11	Trial 1b: open pollination	600	Number of fruits and seeds
SM1219–9		600	
HMC1		600	
**Experiment 2**			
SM1219–9	SM1219-9	30	PTT in 90 ovules
SM1219-9	HMC1	30	PTT in 90 ovules
SM1219-9	Isolation (unpollinated)	10	PTT in 30 ovules
**Experiment 3**			
MNga 11	SM1219-9	70	PTT in 210 ovules per cross
SM1219-9	MCol1505	70	
MCol 1505	MNga 11	70	
**Experiment 4**			
GM1070-17 GM1423-1 GM1458-18 GM1626-20 GM4371-1 GM4377-42 GM4414-5 GM5202-2 HB60 SM3060-34 SM3112-70 SM2629-36 SM3158-26 SM3690-1	GM4414-5 (1), GM4419-16 (65), GM6182-12 (70), and Unknown (1)	16 2 5 29 11 3 7 14 3 11 6 8 4 18	A total of 137 female flowers from which 411 ovules were available for analysis. However, for a more balanced data from only 324 ovules was used for the analysis.
**Experiment 5**			
GM 3692–76	GM 4385–7 (37) GM 4393–14 (26) GM 5270–34 (39) GM 5270–38 (37) GM 5270–7 (41) MCol 638 (52)	23	A total of 232 female flowers were harvested from which 696 ovules could be extracted. Few samples were lost so only 691 ovules were analyzed
GM 3761–29		6	
GM 4385–7		21	
GM 4527–2		17	
GM 5270–6		24	
GM 5270–34		23	
GM 5270–37		28	
GM 5270–38		24	
MCol 1505		22	
MNga11		23	
SM 1219-9		21	

^a^Mixture of flowers of all remainin genotypes available in the field on the pollination day.

### 2.2 Field plots and cultivation practices

Plants were grown in CIAT Experimental Station in Palmira, Valle del Cauca Colombia about 1000 m above sea level and with clay loam soils with a 7.2 pH. Cassava clones were planted following the standard approach based on stem cuttings of approximately 20 cm in length with 5–7 nodes. To facilitate the collection of flowers, stem cuttings were planted with a distance of 2.4 × 0.80 m (a plant density lower than the standard of 10,000 plants per hectare). Irrigation was provided as necessary (in the absence of rainfall, every other week). Fertilization and weed control were the standard for cassava grown at CIAT Experimental Station [27–29]. Occasionally there was a need to apply insecticides to keep whiteflies (*Aleurotrachelus socialis* Bondar) under control.

### 2.3 Pollination protocol

Covering of inflorescences and controlled pollinations were done according to breeding routine applied in CIAT [9]. Female flowers open during the afternoon hours. Early in the morning, experienced field workers identified inflorescences bearing flowers ready for anthesis and covered them with a mesh bag to prevent undesirable pollinations. The remaining flowers in the inflorescence were removed manually. Male flowers were collected early in the morning before their anthesis and placed in plastic bottles with their origin properly identified. They were kept at room temperature until the afternoon when they were used for pollination (their bracts were already opened at that time). Pollination was done by rubbing anthers on stigmas until it was visible that they were covered with pollen (e.g. the stigmas turn yellowish). The isolation bag, removed for the pollination procedure, was immediately placed back and kept protecting the inflorescence until the material was collected for analyses. The collection timing varied among experiments and it is indicated accordingly in section 2.5

### 2.4 Fixation and staining protocol for microscopy study of pollen tube growth within tissue

Upon collection, the flowering stem was cut about 0.5 cm below bracts. The latter were removed and flowers were immediately submerged in the fixation solution (ethanol: acetic acid, 3:1 v/v). The solution was renewed once 24 h after collection. Samples were stored in a refrigerator at 4°C until further processing. When samples had to be preserved for several weeks or longer, they were transferred to 70% ethanol solution for storage.

Preparation of samples for pollen tube growth analysis began with softening and clearing of tissue by submerging samples in 8 N NaOH. Initially (Experiment 2 below), this phase was performed in a hot-water bath of 60°C for 1 h or 12 h at room temperature (overnight). In later experiments, the clearing was performed always at room temperature. Time required for the tissue softening was related to the age of the flowers and the time after pollination: 3–4 h was enough for material fixed until 1 day after anthesis (DAA), while samples collected 2–3 DAA required up to 6 h of clearing. During softening, tissues turned brown-black, probably due to the reaction of secondary metabolites with NaOH in high concentration. The phenomenon does not disturb analysis. Two 1-h washes with a 0.5 N K_2_HPO_4_ buffer solution of pH 10–12 were followed by sample incubation in decolorized 0.1% aniline blue solution in K_3_PO_4._

For dissection, individual pistils were placed on a microscopic slide in a drop of glycerol. Stigmas were cut off from ovaries, flattened on slides and analyzed separately. The three ovarian loculi were separated from each other with dissecting needles, and ovules were then excised. A drop of the aniline blue solution as described above was added to the stigma and the dissected ovules, and then samples were gently squashed under separate coverslips. Slides can be preserved in a horizontal position in 10°C up to several weeks on the condition that their edges were protected from drying by nail polish.

The number of pollen grains on stigma visible after processing and the rate of pollen tube growth through the pistil were assessed using a fluorescence microscope. For experiments 2–4 a Leitz microscope (Wetzlar, Germany) model Aristoplanwith UV illumination was used. Analysis of a part of the experiments 2 and 3 was carried out in Krakow with help of a Nikon Eclipse 600 Fluorescence microscope with UV-2B filter block. Flowers collected in experiments 4 and 5 were analyzed with a Leica Microsystems CMS GmbH (Wetzlar, Germany) DM IL LED microscope using the DAPI filter block. The general anatomy of cassava pistil was dissected using paraffin sections 8-µm thick stained with safranin and fast green processed according to [30].

### 2.5 Experimental designs

#### 2.5.1 Assessment of stigma receptivity by fruit and seed setting (Experiment 1)

Experiment 1 was divided into two trials. In the first trial (1a), similar number of female cyathia from MNga11, SM1219-9 or a bulk from different available genotypes (Table 1) were randomly assigned to one of the following treatments: **a)** no coverage of cyathia, open pollination; **b**) the coverage of cyathia as described in 2.3 but mesh bags were removed one day after anthesis (1 DAA); **c)** the coverage was removed 2 DAA and **d**) mesh bags were removed 3 DAA. After removing the isolation bags, flowers were left exposed for open pollination. Each treatment was marked with a tag with the required information (the date of the coverage and the coverage removal and the number of flowers treated in the particular inflorescence). The fruit set for each treatment was established 60 DAA.

The second trial (1b) was similar to the first one (Table 1). The main difference was that fruits were covered again with mesh bags as they approached maturity, so seeds could be collected and counted upon dehiscence (standard procedure in CIAT). A total of 150 flowers per female genotype were randomly assigned to one of four treatments (keeping flowers covered for 0, 1, 2 or 3 DAA).

#### 2.5.2 Microscope study of stigma receptivity and pollen tube growth (Experiment 2)

The goal of this experiment was to establish the period of stigma receptivity in relation to bracts opening and the effect of early or delayed pollination on the pollen tube growth as well as the possible presence of post-pollination crossing barriers. The efficiency of the isolation system with a mash bag performed as described in 2.3 was additionally assessed.

Seventy female flowers from clone SM 1219-9 were isolated from unwanted pollination 1–2 days before anthesis. Flowers remained covered until the collection of pistils (except for the pollination procedures, when appropriate). As a control of isolation procedures, a random sample of 10 of these flowers were isolated, left unpollinated, then collected, and fixed three DAA. The remaining 60 flowers were either self-pollinated or crossed with pollen from genotype HMC1 (30 flowers per type of cross) (Table 1). The flowers for each type of cross were equally distributed and pollinated at five different times in relation to anthesis: **1)** pollination of unopened flowers by forcing bracts to expose pistil one day before anthesis; **2)** pollination on the anthesis day, i.e. on the day bracts naturally open; **3)** pollination 1 DAA; **4)** pollination 2 DAA; and **5)** pollination 3 DAA. This means that six flowers per type of cross were pollinated at each given time. Samples were collected and fixed 24, 48 or 72 h after pollination (HAP) and stained with aniline blue as described in 2.4. This means that the sample size for each type of cross, pollination time and collection time was two flowers (six ovules).

**Figure 1.**
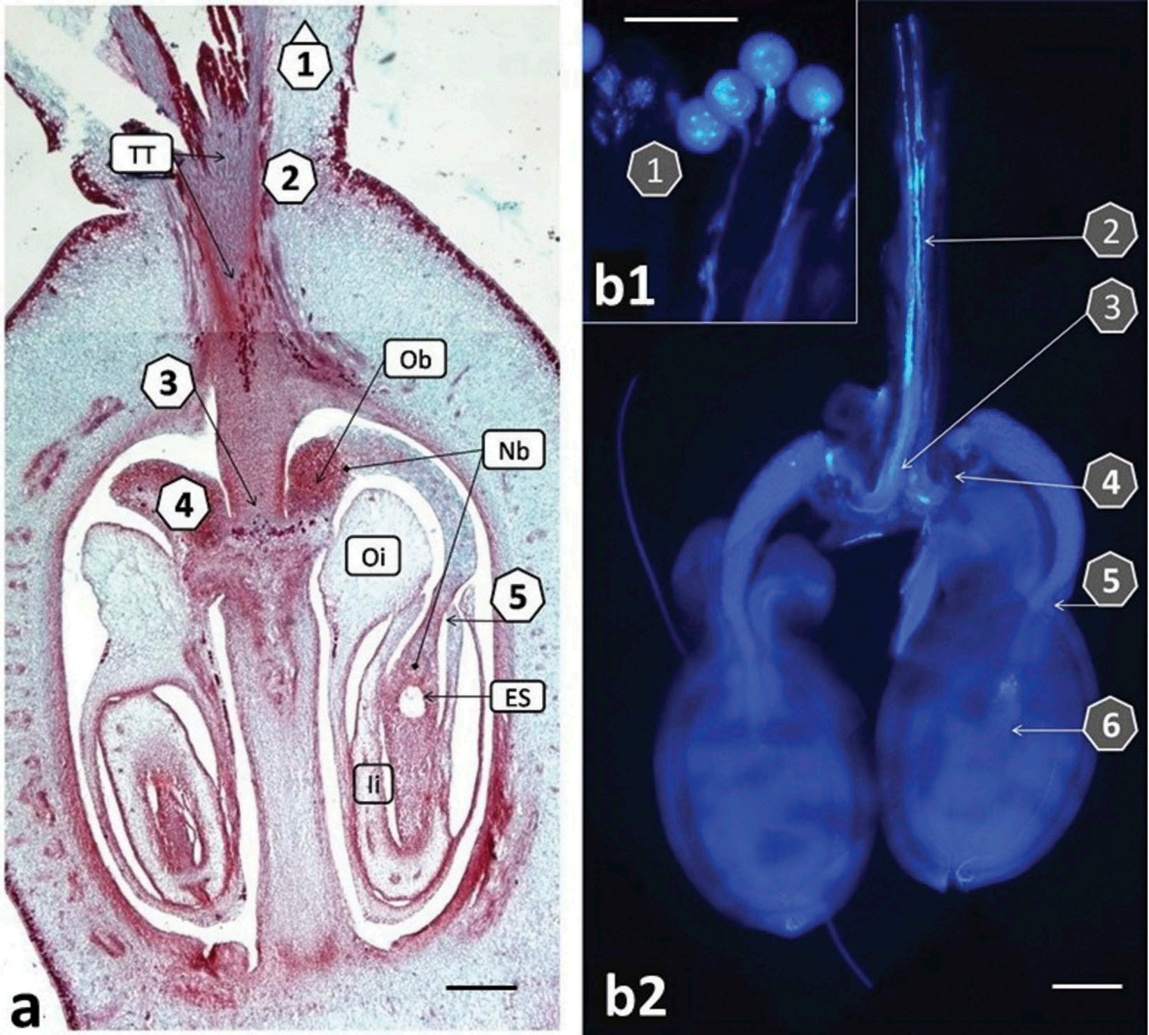
Cassava (*Manihot esculenta* Cranz). Visualization of pollen tube pathway from stigma to embryo sac. a. Crossection of the cassava pistil (paraffin section 8 µm thick stained with safranin and fast green). b. The same parts of pistil as visible on A shown on preparation of ovules dissected from the pistil and stained with aniline blue, analyzed under UV light. Labels 1–6 show the main checkpoints for pollen tube growth analysis [1]. PTT on the stigma surface or its shallow ingrowth into the transmitting tissue (TT) (a, b1) [2]; PTT in the transmitting tissue of the style (a, b2) [3]; PTT at the bottom of the style (a, b2) [4]; PTT in the obturator (Ob) (a, b2) [5]; PTT in the nucellar beak (Nb), (a, b2); and [6] PTT at the embryo sac (ES), (a, b2). Outer (Oi) and inner (Ii) integuments of the ovules are also visible. Pictures were composed of several images ensemble together. Magnification A, B2 10x, B1 20x (scale bar = 200 μm).

Two response variables were considered in this study: score on the number of pollen grains that could be counted on the stigma surface and the furthermost position of the longest pollen tube tip (PTT) 24, 48 and 72 HAP. The score for assessing the quantity of pollen grains on the stigma surface had four categories: 1: No pollen grains; 2: from 1 to 5 grains; 3: from 6 to 24 grains; and 4: 25 or more pollen grains. The staining procedure is harsh; therefore, the number of PT staying on the stigma after the last washing is equal or very close to the number of pollen grains that effectively germinated on the stigma and produced at least short PT. Six milestone sites were checked for the presence of PTT as follows (Figure 1): 1) PTT on the stigma surface or a shallow ingrowth to the transmitting tissue; 2) PTT in the transmitting tissue of the stigma; 3) PTT at the bottom of the style but not yet in the obturator; 4) PTT in the obturator; 5) PTT in the nucellar beak but not further than half of its length; and 6) PTT passed over half of the beak visible at the vicinity of the embryo sac.

#### 2.5.3 Pollen tube growth rate in cassava crosses (Experiment 3)

The purpose of this experiment was to establish the rate of pollen tube growth and possible time of fertilization as in Experiment 2. However, pistils were collected at additional time points after pollination and in wider genetic backgrounds. Crosses were made between MNga11 x SM1219-9; SM1219-9 x MCol1505; and MCol1505 x MNga11 (Table 1). Pollination was performed as described in section 2.3. Results from Experiment 2 prompted to expand the number of sampling times and to concentrate on PT growth from the nucellar beak to the embryo sac. Samples of 10 flowers were collected 6, 13, 18, 26, 42, 66, and 74 HAP for each cross (70 flowers per cross). The nucellar beak is considerably long. Therefore, additional information was taken regarding how far through the entire length of the beak the pollen tubes had advanced (e.g. 1/8, 1/4, 1/2, and 3/4 of its length from the tip).

#### 2.5.4 Pollen tube growth in a wide genetic background (Experiment 4)

Flowers from plants growing in a crossing nursery for the cassava-breeding project were used (Table 1). Pollinations were done with three sources of pollen: GM4414-5 (used in just one pollination), GM4419-16 (65 pollinations) and GM6182-12 (70 pollinations). Pollinated flowers were harvested at 16, 48, 72 and 96 HAP. Pollinations were performed as described in section 2.3 and flower collections and processing as described in section 2.4. Milestones to assess PT growth were the same as for Experiment 3 (section 2.5.3).

#### 2.5.5 Assessing rate of pollen tube growth in wider set of genotypes (Experiment 5)

This experiment is similar to the previous one, except that a different set of progenitors were used and pollinations were made in a different time of the year (Figure 4). Experiment 5 was also done in the ordinary crossing nursery from the cassava-breeding project. Eleven elite clones were used as female progenitors whereas six clones were used as a source of pollen. Three genotypes were used both as male and as female progenitors (Table 1). Flower collection, sample preparation and analysis were done at two different time points: 72 and 96 HAP. Milestones to assess PT growth were the same as in Experiment 3 (section 2.5.3).

### 2.6 Statistical analysis

Analysis of variance was made using the GLM procedure in SAS [31]. Statistical significance for differences between means was determined using the Least Squares Difference (LSD) approach provided by the GLM procedure.

## 3. Results

### 3.1 Visualization of the pistil anatomy and pollen tubes within pistil

Cassava pistils contain three ovarian loculi each containing one anatropous, crassinucellar ovule. Many details of cassava pistil anatomy are typical only for *Euphorbiaceae* (Figure 1). The stigma is thick and its three lobs fuse into short style (Figure 1(a)). Transmitting tissue facilitates the PT passage. It is formed in the stigma and continues through the style. The PT track goes further into a specific outgrowth of ovarian columella – the obturator. Nucellus of the ovule forms a long, thick outgrowth (the nucellar beak), which protrudes from the integuments and reaches the obturator. Relatively thick integuments surround ovules. At the end of the outer integument, there is a widening called elaiosome or caruncle. The typical micropyle is not present in cassava. The narrowest section of the nucellar beak between the integument edges will be called pseudomicropyle in this article (checkpoint 5 in Figure 1). The embryo sac is large and clearly visible even under a low magnification microscope (Figure 1).

**Figure 2.**
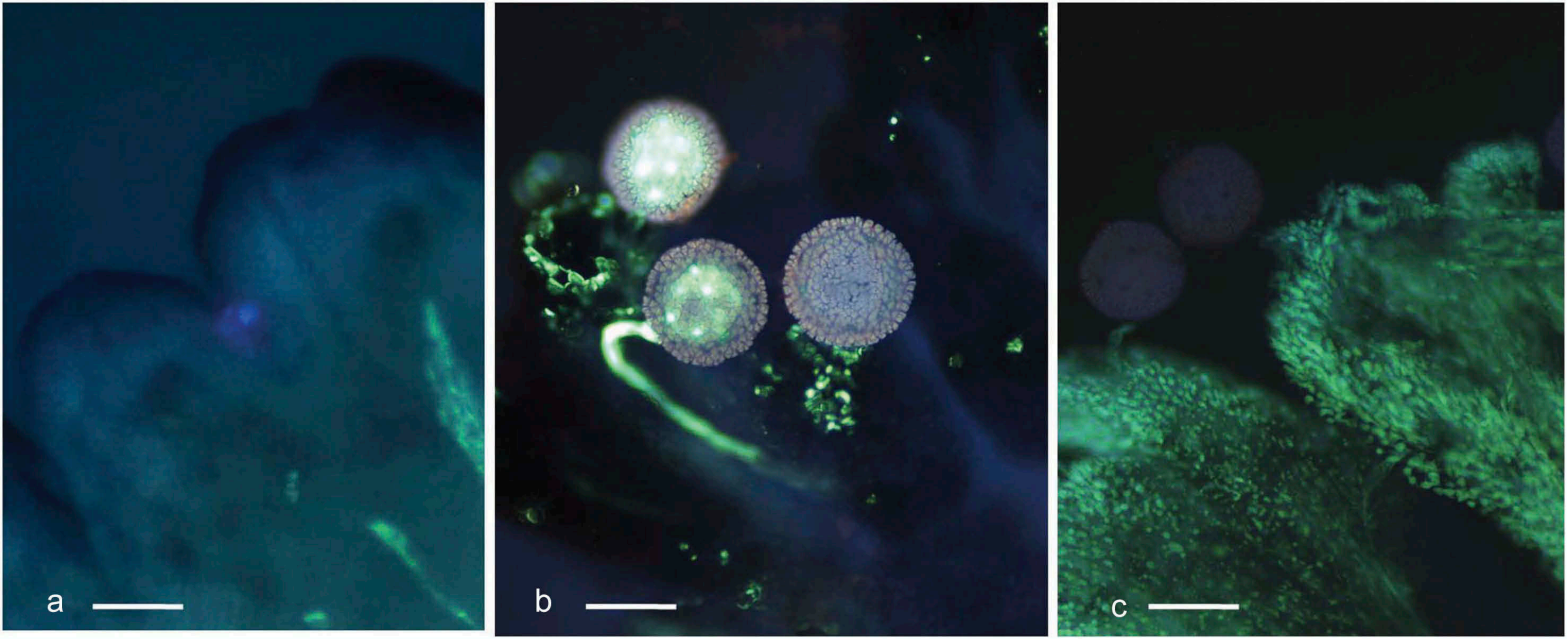
Cassava (*Manihot esculenta* Cranz) stigmas stained with aniline blue and analyzed with UV, wideband filter. a. Stigma in freshly exposed flower. Stigma’s edges are dark blue. Vascular bundles visible on the right lower corner show pale fluorescence. b. Germinating pollen on the stigma. One pollen tube shallow ingrowth to the stigma is visible in the center. Stigmatic tissue gives bright callose reaction in the vicinity of pollen grains. c. Stigma fixed three days after pollination. Fluorescence of two grains and their tubes has faded. Stigma surface gives strong yellow fluorescence typical for a fading process (scale bar = 100 μm).

**Figure 3.**
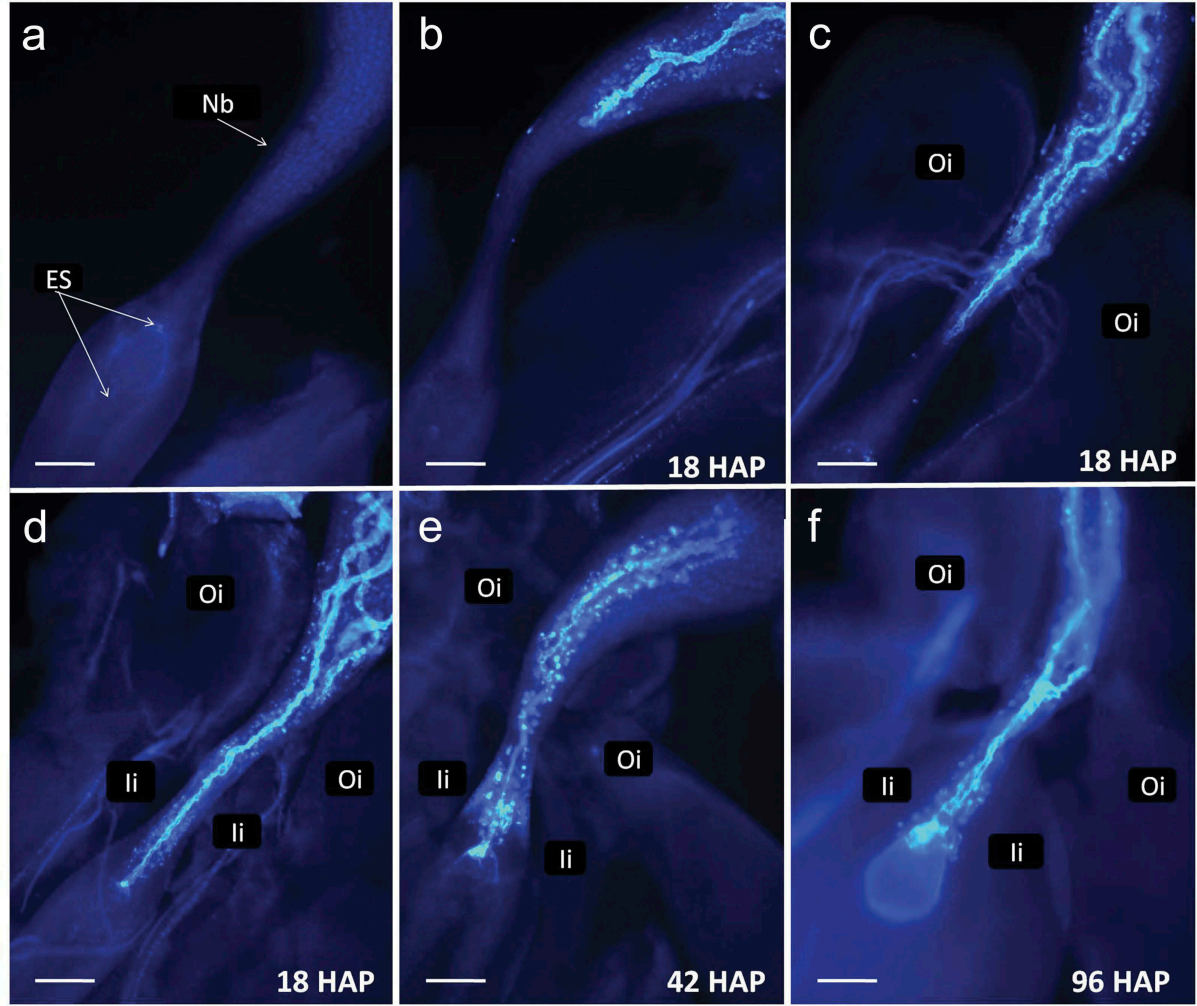
Cassava (*Manihot esculenta* Cranz). Series of pictures from dissected ovules stained at different time points to demonstrate pollen tube passage through the ovule. a. An ovule dissected from a flower at time of bracts opening. Integuments were removed. Nucellus forms large beak (Nb). **b-**d. Fragments of ovules dissected 18 h after pollination (HAP) showing variability of pollen tubes length. Several tubes are visible on c and d. The dominating pollen tube reaches vicinity of the embryo sac on d. Callose reaction is visible in beak after pollen tubes passage. e. Ovule fixed 42 HAP with pollen tube entering the egg apparatus. f. A pollen tube entered the egg apparatus giving a strong fluorescence in one of the cells. The second pollen tube stopped and swollen at the narrowest part of the beak, so-called pseudomicropyle. Oi – outer integument, Ii – inner integument (scale bar = 100 μm).

The staining with aniline blue, which reacts with and highlights callose, facilitated tracking pollen tube growth throughout the pistil in the stigma, the style and into the ovule (Figures 2 and 3).The callose reaction in tissues after pollen tubes passage is clearly visible in Figures 2 and 3. It can be seen as fluorescing rings surrounding pollen tubes on the stigma surface (Figure 2(b)) and fluorescing dots within the beak (Figure 3). The very strong fluorescence is visible within the embryo sac after the pollen tube entrance to the egg apparatus.

Aniline blue and UV light incite also no-callose specific fluorescence on the surface of wilting stigma (Figure 2(c)). Intensity of fluorescence tends to be negligible in fresh stigma/style tissue (Figure 2(a)), but it was considerably strong when the tissue was wilting because of its aging and/or because of pollen tubes penetration. It was possible, therefore, to assess qualitatively the appearance of the stigma (e.g. fresh or old-looking). This information was then used to relate the appearance of stigma/style tissue with results on pollen germination and tube growth in some of the experiments. Non-callose specific fluorescence was also visible in the vascular bundles of the integuments (Figure 3(b-e)). The vascular bundles fluorescence, however, was weaker in comparison to that of pollen tubes.

**Figure 4.**
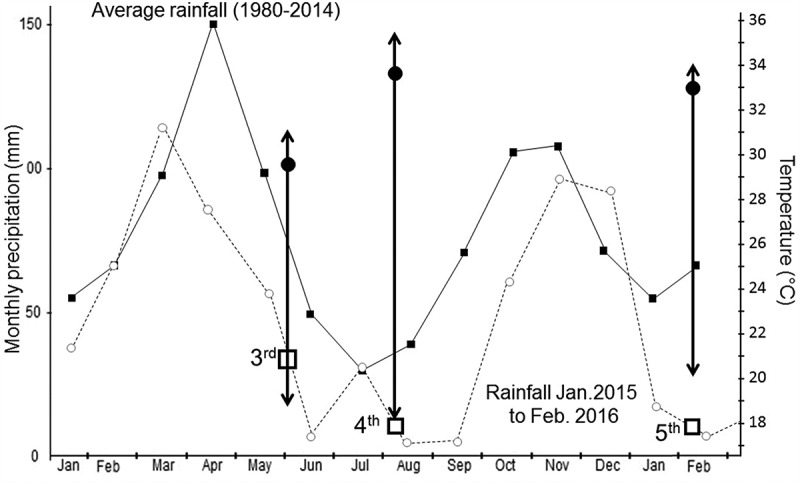
Average monthly rainfall in the 1980–2014 period and during the period in which *Experiments 3–5* were conducted. Boxes indicate the duration of each experiment. Arrows indicate the range of variation (minimum to maximum) temperatures, respectively, for each experiment. Black circles indicate average temperature along each experiment.

The studies described in this article were conducted through several years. Early results suggested two unexpected trends in pollen tube growth: the surprisingly long time needed for PT to reach embryo sac, which additionally varied widely from one cross to another. Therefore, several successive experiments were carried out that included the collection of flowers up to 96 HAP and at different time intervals. Together the experiments provide a large sample of ovules from different genotypes, pollinated with different sources of pollen, and collected at different periods during the year under varying environmental conditions (as illustrated in Figure 4).

### 3.2 Assessment of stigma receptivity by fruit and seed setting

**Figure 5.**
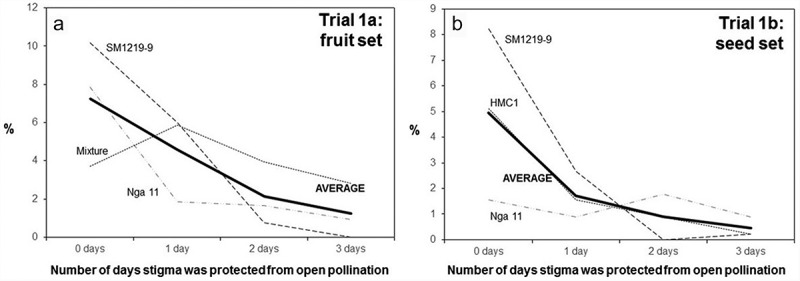
Fruit (a) and seed (b) set observed from female cyathia that were subjected to different treatments: flowers not covered (0 days) or covered for 1, 2 or 3 days after anthesis (horizontal axis). The two independent trials were carried out to assess the duration of stigma receptivity. The vertical axis in the left plot presents the number of fruits per flower counted in the first trial. The vertical axis in the plot on the right presents the actual number of seeds in relation to the expected number based on the total number of flowers considered (three seeds per pistil). SM1219–9, Nga 11, HMC1 are cassava clones, Mixture – represent numerous other clones available in the field when Trial 1a began. AVERAGE represents the mean value of three types of genotype in each trial.

The two trials of Experiment 1 (Table 1) were conducted to assess for how long after anthesis stigmas remain receptive in cassava. Figure 5 summarizes the results of the two trials. The vertical axes in the plots of this figure express the percentage of flowers that resulted in fruit (Figure 5(a)) or seeds (Figure 5(b)) expected based on the number of flowers considered in each trial. Since cassava ovaries have three loculi with ovules, up to three seeds per flower/fruit were expected.

The second trial was actually implemented as an evolution of the first one, which had suggested that fruit set may be a misleading response variable because of the occasional occurrence of parthenocarpic seedless fruits. Based on both trials it can be concluded that stigmas remain receptive for up to three days after bracts opening (anthesis) and that there is large variation in reproductive efficiency of different genotypes (Figure 5). Fruit and seed set from open pollination is well below the maximum potentials (about 7% in case of fruit and 5% of expected seed, as illustrated, respectively, in Figure 5(a,b)).

### 3.3 Microscope study on stigma receptivity in cassava (Experiment 2)

Results of the second experiment allowed for the analysis of variance presented in Table 2. As expected, no pollen grains or tubes were observed on stigmas of 10 flowers left unpollinated and covered for three days until collection. Results from these flowers will not be further discussed.

**Table 2.** Analysis of variance for data from Experiment 2 in which pistils from SM 1219-9 were self- or cross-pollinatinated with HMC1 (30 flowers for each type of cross yielding a total of 180 ovules). Pollinations were made one day before anthesis, on the anthesis day or 1, 2, or 3 days after anthesis (Pollination time). Flowers were collected 1, 2, or 3 days after pollination (Collection time). Response variables were: the number of pollen grains counted on the surface of the stigma and the position of the furthermost pollen tube tip within the pistil.

Source of variation	df	Mean squares^a^	
		No of pollen grains per stigma	Position of pollen tube tip in the pistil^b^
Self pollination vs cross (T)	1	0.568	6.349
Pollination time (P)	4	2.285***	91.125***
Collection time (C)	2	7.340***	7.107**
T*P	4	5.275***	3.946
T*C	2	0.312	3.255
T*P*C	16	1.902***	10.318***
Error	146	0.482	2.313

^a^***, **Significant at P < 0.01, and P < 0.05 probability level, respectively.

^b^Furthermost position of the pollen tube tip (PTT) was assessed at different anatomical points of the pistil. Score was based on the pistil anatomy as follows: 1 – PTT on the stigma surface or a shallow ingrowth to the transmitting tissue; 2 – PTT in the transmitting tissue of the style; 3 – PTT at the bottom of the style; 4 – PTT in obturator; 5 – PTT in the nucellar beak; and 6 – PTT at the vicinity of the embryo sac.

There were three main sources of variation, namely the contrast between self-pollinations and crosses, time of pollination in relation to anthesis and harvesting time after pollination. The statistical significance of the interaction among some of these sources of variation was also assessed. There was no significant difference for density of pollen grains counted on the surface of the stigma nor on the depth of the pollen tube growth when the type of pollination was considered (self- versus cross-pollinations). This is important and validates empirical evidence that self-pollinations do not offer any particular problem apart from the lack of synchronization for male and female cyathia. Pollination time, on the other hand, significantly (P < 0.01) affected pollen density and pollen tube length (Table 2). Collecting time also had a significant effect on pollen grain density (P < 0.01) and depth of pollen tube growth (P < 0.05).

The implication of this finding can be clearly understood by looking at Table 3, which presents the actual averages for each of these two response variables. Data presented in this table was pooled across the two types of crosses considering that there were no significant differences among them (Table 2). It is acknowledged that some of the interactions involving the type of cross reached statistical significance. However, pooling the data facilitates drawing some of the general trends that this study sought for. The highest averages for pollen density and depth of PTT growth were observed in flowers pollinated on anthesis day (Table 3). The second-best results were observed in pollinations made the day before anthesis (particularly in relation to pollen tube growth). Results for pollinations made after anthesis day were not consistent but always significantly worse than earlier pollinations.

**Table 3.** Results from the second experiment. Assesment of pollen grains number on stigma after clearing and staining with aniline blue and the furthermost pollen tube tip position in pistil in samples collected at a different time in relation to anthesis and on a different number of days after pollination. Pooled data of SM 1219–1 crossed with HMC-1 or self-pollinated. A total of 60 flowers (180 ovules) were analyzed^a.^

Pollination of stigma in relation to anthesis (days)	Collection time after pollination (hours)	Pollen grains number on stigma after aniline blue staining^b^	Position in pistil of the furthermost pollen tube tip^c^
One day before anthesis (-1)	24	1.25e	2.33cde
	48	2.67ab	3.67bc
	72	3.00a	5.25a
Anthesis day (0)	24	2.58abc	4.50ab
	48	2.50abc	5.25a
	72	2.75ab	4.83ab
One day after anthesis (1)	24	1.58de	2.00def
	48	1.55de	1.64ef
	72	2.75ab	1.08efg
Two days after anthesis (2)	24	1.58de	0.92fg
	48	2.50abc	0.08g
	72	2.00cd	2.08def
Three days after anthesis (3)	24	2.25bc	3.08cd
	48	2.25bc	1.33efg
	72	2.27bc	2.00def
-1	Averaged across all collection times in relation to pollination day	2.26b	3.75b
0		2.61a	4.86a
1		1.97b	1.57cd
2		2.03b	1.03d
3		2.26b	2.14c
Averaged across all pollination times in relation to anthesis day	24	1.85b	2.57ab
	48	2.3a	2.40b
	72	2.5a	3.07a

^a^For each section of the Table, values with the same letter within each column were not statistically different at P < 0.05, based on the LSD test.

^b^Scoring for pollen grain number was as follows: 1 – No pollen grains; 2 – from 1 to 5 grains; 3 – from 6 to 24 grains; and 4 – 25 or more pollen grains.

^c^Average position of the furthermost pollen tube tip (PTT) was assessed at different anatomical points of the pistil. Score was based on the pistil anatomy as follows: 1-PTT at the stigma surface or its shallow growth into the transmitting tissue; 2-PTT in the transmitting tissue of the style; 3-PTT at the bottom of the style; 4-PTT in the obturator; 5-PTT in the nucellar beak; and 6-PTT at the vicinity of the embryo sac.

Pollen density was significantly higher in flowers harvested 2 or 3 DAP (average scores of 2.31 and 2.54) compared with those harvested just one day after pollination (average score of 1.85). For pollen tube growth, results were not consistent, but the highest progress was observed in flowers harvested 3 days after pollination (bottom of Table 3). It is important to report that in 26 cases pollen tubes reaching the embryo sac had been observed. Of those, 9, 7 and 10 cases came from flowers harvested 1, 2, and 3 DAP, respectively. Since a total of 180 ovules were analyzed this means that cumulatively in 5%, 9% and 14% of the cases the pollen tubes had reached the embryo sac, respectively, 1, 2 and 3 DAP. The progress of pollen tubes, however, did not show a consistent pattern of a closer approach to the embryo sac in later collections of flowers. The average positions of the PTT were, respectively, 2.57; 2.40 and 3.07, respectively, for flowers collected 1, 2 or 3 days after pollination (Table 3).

### 3.4 Pollen tube growth rate in cassava crosses (Experiment 3)

A total of 210 flowers were collected, resulting in 630 ovules for analysis. Of those only four samples could not be analyzed (because of problems during sample preparation): SM1219-9 (6 HAP); SM1219-9 (66 HAP); MNga 11 (66 HAP); and MCol 1505 (74 HAP). However, the dataset can be considered balanced, in spite of these few missing data. The analysis of variance focused on the effect of the female progenitor, time of flower collection after pollination and the interaction of these two sources of variation (Table 4). All these sources of variation were statistically significant (P < 0.01). The significant effect of female progenitor is validated by the respective averages also presented in Table 4. Across all the collecting times in about 70% of samples from SM1219-9, PTT had reached (or grown beyond) the nucellar beak. In the case of MNga11 and MCol 1505, the averages were 57% and 48%, respectively (Table 4). The averages for each progenitor are also useful to understand the slow progression of the pollen tube towards the embryo sac once they have reached the nucellar beak. In about 43% of all samples from SM1219-9, PTT reached the pseudomicropyle (e.g. end of the nucellar beak) and in only 10% had reached the embryo sac. In the case of MNga11 and MCol 1505, they both had about 25% of samples with PT at the pseudomicropyle or beyond. However, in the case of MNga 11 only in 2% of the samples the PTT had reached the embryo sac, whereas in the case of MCol 1505 the average was significantly higher (8%).

**Table 4.** Relevant results from Experiment 3. Analysis of variance and proportion for the presence of pollen tube growth ^(a)^ after controlled pollinations in three cassava female parents: SM 1219-9, MNga 11 and MCol 1505. A total of 210 pistils (630 ovules) were analyzed. The source of pollen was the same three genotypes. Pollinated pistils were harvested at seven different times ranging from 6 to 74 h after pollination (HAP).

		Nucellar beak		Micropylar	Embryo
Source of variation	df	Proportion^(a)^	depth^(b)^	region^(a)^	Sac^(a)^
**Analysis of variance**					
Female parent (F)	2	2.752***	3.172***	2.232***	0.334***
Collection time (C)	6	2.470***	3.333***	7.856***	0.848***
F*C	12	0.624***	0.374***	0.475***	0.272***
Error	605	0.205	0.147	0.126	0.047
**Average for each female parent^(c)^**					
SM1219-9		0.707a	0.600a	0.428a	0.096a
MNga 11		0.569b	0.440b	0.254b	0.019b
MCol 1505		0.478c	0.357c	0.244b	0.077a
**Average pollen tube growth for samples collected at different HAP**^(d)^					
6 HAP		0.449b	0.171d	0.000f	0.000d
13 HAP		0.822a	0.535b	0.000f	0.000d
18 HAP		0.511b	0.378c	0.122e	0.000d
26 HAP		0.433b	0.362c	0.267d	0.022cd
42 HAP		0.433b	0.417c	0.400c	0.067bc
66 HAP		0.693a	0.668a	0.636b	0.091b
74 HAP		0.753a	0.734a	0.742a	0.270a
**Least square means for stigma/style quality (only 74 HAP)**^(e)^					
Fresh		0.768a	0.748a	0.753a	0.301a
Deteriorated		0.645a	0.634a	0.661a	0.066b

*** Significant at P < 0.01 probability level.

^(a)^Position of the furthermost pollen tube tip (PTT) was assessed in the pistil and score was based on the pistil anatomy: nucellar beak; PTT the micropylar region of the beak; and PTT in vicinity of the embryo sac. These variables relate to the proportion of pollen tubes at each of these milestones for pollen tube growth.

^(b)^Proportion of the total length of the nucellar beak covered, on average, by the growth of the pollen tubes.

^(c)(d)(e)^ For each section of the Table, values with the same letter within each column were not statistically different at P < 0.05 (based on the LSD test).

Table 4 also presents the averages (across the three female progenitors) for each collection time. A large proportion of pollen tubes (>82%) reached the nucellar beak as early as 13 HAP. A consistent progress of PTT, in relation to the collection time, can be clearly observed in the last two columns of Table 4. As early as 18 HAP the first pollen tubes were observed reaching the pseudomicropyle. By 74 HAP the percentage of samples in which the pollen tubes had reached the pseudomicropyle region was 74%. Pollen tubes could be observed reaching the embryo sac for the first time at 26 HAP (2% of samples). As expected, the number of samples in which pollen tubes had reached the embryo sac increases gradually with time: 7% of the samples 42 HAP, 9% of the samples 66 HAP, with a significant increase at 74 HAP (27%). These results are consistent with those from the previous experiment regarding the slow growth of pollen tubes (particularly beyond the pseudomicropyle) and the considerable variation that can be observed. As the tip of the pollen tube reaches the end of the nucellar beak (pseudomicropyle) or beyond, the trail left behind (distal portion of the nucellar beak) becomes less reliable. Perhaps this is because the PTT had gone through the nucellar beak too many hours before the collection of the samples. This observation may explain why the proportion of samples with PTT in the nucellar beak is not consistent in samples collected after 13 HAP.

The effect of female flower quality (e.g. the age of the respective pistil) on pollen tube growth can be assessed from data presented at the bottom of Table 4. Although in every case pollen tube growth was slower in aged flowers compared with fresh ones, differences did not reach statistical significance (data not presented). The differences were particularly large and reached statistical significance only when comparing pollen tubes at the embryo sac in samples collected at 74 HAP. This was the only case where the average from fresh tissue (0.301) was significantly higher than in aged flowers (0.066) at P < 0.01. This is why only data from 74 HAP is presented in Table 4. Figure 3 presents examples of pollen tubes reaching different milestones within the pistil. It is interesting to note that several pollen tubes can reach the nucellar beak but only one of them would continue growing beyond the end of it.

### 3.5 Assessing rate of pollen tube growth in wider set of genotypes (experiment 4)

The main objective of this trial was to validate the results from the previous experiment using data from a different set of female progenitors (up to 14) compared with all the work conducted earlier which used small and repeated set of clones. It also included, for the first time, the collection of flowers 96 HAP hoping to see a higher proportion of pollen tubes reaching the embryo sac than the 27% observed in the previous Experiment 3. The number of loculi available for different harvesting times was not uniform (81, 114, 123, and 93 loculi, respectively, for harvests at 16, 48, 72 and 96 HAP). Because of the large lack of balance in the number of loculi available for each harvesting time, data from several loculi (particularly those collected 48 and 72 HAP) were randomly ignored from the analysis (Table 1). This process was done in such a way to maximize the balance of the data.

The results of the analysis of variance are at the top of Table 5. The timing of flower collection (in relation to pollination time) resulted in significant differences for pollen tubes at the nucellar beak (P < 0.10) and pseudomicropyle (P < 0.05) but not at the embryo sac. In general, this experiment produced less consistent results compared with the previous one, which in part may be due to the smaller number of samples from each female progenitor. In every case, as expected, values for flowers collected 96 HAP were always significantly higher than samples collected earlier (except for PTT at the embryo sac, which was similar 48 and 96 HAP). On the other hand, surprisingly, data from flowers collected at 72 HAP were lower than those from the 48 HAP treatment.

**Table 5.** Analysis of variance and relevant averages for pollen tube growth in controlled pollinations in 14 cassava female parents in Experiment 4. A total of 108 pistils (324 ovules) were analyzed in this study. Pollinations were made using three male progenitors. In one case the tag identifying the source of pollen was missing. Pistils were harvested at four different times ranging from 16 to 96 h after pollination (HAP).

		Nucellar beak			
Source of variation	df	Proportion^(1)^	Depth^(2)^	Micropylar region^(1)^	Embryo sac^(1)^
**Analysis of variance**					
Female parent (M)	13	0.672***	0.529***	0.428***	0.119
Hours (H)	3	0.475*	0.170	0.402**	0.160
M*H	26	0.486***	0.374***	0.305***	0.104
Error	280	0.186	0.148	0.145	0.083
**Average pollen tube growth for samples collected at different HAP**^(3)^					
16 HAP		0.413ab	0.263b	0.100c	0.025b
48 HAP		0.333 bc	0.307ab	0.247ab	0.148a
72 HAP		0.247c	0.225b	0.173bc	0.062b
96 HAP		0.469a	0.423a	0.358a	0.148a
**Least square means for stigma/style quality (96 HAP)**^(4)^					
Fresh		0.438a	0.388a	0.335a	0.143a
Deteriorated		0.631a	0.377a	0.000b	0.016a

***, **, * Significant at P < 0.01, P < 0.05 and P < 0.10 probability level, respectively

^(1)^Position of the furthermost pollen tube tip (PTT) was assessed in the pistil and score was based on the pistil anatomy: nucellar beak; PTT the micropylar region of the beak; and PTT in vicinity of the embryo sac. These variables relate to the proportion of pollen tubes at each of these milestones for pollen tube growth.

^(2)^Proportion of the total length of the nucellar beak covered, on average, by the growth of the pollen tubes.

^(3)(4)^For each section of the Table values with the same letter within each column were not statistically different at P < 0.05 (based on the LSD test).

Contrary to the expectations, the average number of pollen tubes observed at the embryo sac 96 HAP (15%, Table 5) was considerably lower than those observed 72 HAP (27%, Table 4) in the previous experiment. One feasible explanation is that a different set of female progenitors had been used. However, perhaps more relevant were the contrasting environmental conditions in which the two experiments were conducted (Figure 4).

### 3.6 Assessing rate of pollen tube growth in wider set of genotypes (Experiment 5)

The last conducted study focused on later stages in the pollination and fertilization process by collecting flowers at 72 and 96 HAP exclusively. Table 6 presents the analysis of variance, which indicates significant effects for male and female progenitors but not for the timing of flower collection. Interaction between timing of harvest with female and female*male sources of variation was significant at varying probability levels.

**Table 6.** Analysis of variance and relevant averages for pollen tube growth in controlled pollinations in crosses between 11 female and 6 male cassava progenitors in Experiment 5. A total of 210 pistils (630 ovules) were analyzed in this study. Pistils were harvested at 72 or 96 h after pollination (HAP).

		Nucellar beak			
Source of variation	df	Proportion^(1)^	depth^(2)^	Micropylar region^(1)^	Embryo sac^(1)^
**Analysis of variance**					
Female (F)	10	5.383***	5.406***	5.232***	2.117***
Male (M)	5	3.700***	2.583***	3.340***	0.476***
Hours (H)	1	0.020	0.062	0.032	0.163
F*H	10	0.251*	0.376***	0.322**	0.316***
M*F*H	75	0.359***	0.391***	0.371***	0.217***
Error	589	0.120	0.117	0.123	0.115
**Average pollen tube growth in samples collected at different HAP^(3)^**					
72 HAP		0.495a	0.477a	0.479a	0.226a
96 HAP		0.487a	0.452a	0.469a	0.181a
**Least square means for stigma/style quality (96 HAP)**					
Fresh		0.500a	0.486a	0.486a	0.192a
Deteriorated		0.368b**	0.340b***	0.312b***	0.188b*

***, **, *Significant at P < 0.01, P < 0.05 and P < 0.10 probability level, respectively

^(1)^Position of the furthermost pollen tube tip (PTT) was assessed in the pistil and score was based on the pistil anatomy: nucellar beak; PTT the micropylar region of the beak; and PTT in vicinity of the embryo sac. These variables relate to the proportion of pollen tubes at each of these milestones for pollen tube growth.

^(2)^Proportion of the total length of the nucellar beak covered, on average, by the growth of the pollen tubes.

^(3)^Values with the same letter within each column were not statistically different at P < 0.05 (based on the LSD test).

As in the previous experiments, there were no major differences (ranging all around 40–50%) for the first three variables (PTT at the beak, the beak depth, and at the pseudomicropyle) but a sharp reduction in the proportions of PTT at the embryo sac (18–22%). No significant differences were observed between samples collected 72 and 96 HAP. On the other hand, significant differences could be observed for flowers whose stigmas looked fresh compared with those with an aged-looking appearance.

The percentage of flowers without stigma (data not presented) increased sharply with the time of flower collection (14.6% to 42.3% respectively for 72 to 96 HAP). Similarly, the proportion of aged-looking stigmas increased from 14.7% to 18.7% in flowers harvested 72 and 96 HAP, respectively.

As stated above, female and male progenitors had a highly significant (P < 0.01) effect on all response variables analyzed (Table 6). The averages across female or male progenitors are presented in Table 7. They illustrate the drastic variation, particularly among female progenitors, ranging from no sample with pollen tubes at the embryo sacs for GM4385-7 to 58% in the case of GM3692-76. The rankings for female progenitors across the four considered variables were rather consistent: GM3692-76 was always the best, independent of the variable considered. Similarly, GM4385-7 was always the worst. Results of the reproductive capacity of different genotypes, when used as a source of pollen, showed significance in areas closer to the stigma, but not at the embryo sac. Pollen from MCol 638 and GM4393-14 showed a better performance compared with those from other genotypes.

**Table 7.** Average depth of pollen tube growth in the pistils of 11 female cassava progenitors and 6 male progenitors (across two harvesting times: 72 and 96 h after pollination) from Experiment 6.

		Nucellar beak			
Genotype	n	Proportion^(1)^	Depth^(2)^	Micropylar region^(1)^	Embryo sac^(1)^
**Mother**^(3)^					
GM 3692-76	69	0.884a	0.884a	0.884a	0.580a
SM 1219-9	62	0.839ab	0.830ab	0.806ab	0.452ab
GM 3761-29	18	0.778ab	0.778ab	0.778ab	0.333bc
MCol 1505	66	0.712bc	0.692bc	0.682bc	0.212cd
MNga11	69	0.594cd	0.572cd	0.580cd	0.188de
GM 5270-37	82	0.512de	0.390ef	0.512de	0.073ef
GM 4527-2	51	0.510de	0.466de	0.431ef	0.196de
GM 5270-34	69	0.406ef	0.396ef	0.391ef	0.159de
GM 5270-38	70	0.329f	0.288f	0.300f	0.157de
GM 5270-6	72	0.042g	0.042g	0.042g	0.000f
GM 4385-7	63	0.032g	0.032g	0.032g	0.000f
**Father**^(4)^					
MCol 638	154	0.636a	0.588ab	0.610a	0.162a
GM 4393-14	78	0.615a	0.515ab	0.590a	0.218a
GM 5270-38	111	0.477bc	0.474bcd	0.468bc	0.234a
GM 4385-7	110	0.427bc	0.423bcd	0.418bc	0.227a
GM 5270-7	125	0.416bc	0.39cd	0.384bc	0.176a
GM 5270-34	113	0.363c	0.363d	0.36c	0.212a

^(1)^ Position of the furthermost pollen tube tip (PTT) was assessed in the pistil and score was based on the pistil anatomy: nucellar beak; PTT the micropylar region of the beak; and PTT in vicinity of the embryo sac. These variables relate to the proportion of pollen tubes at each of these milestones for pollen tube growth.

^(2)^ Proportion of the total length of the nucellar beak covered, on average, by the growth of the pollen tubes.

^(3)(4)^ For each section of the Table values with the same letter within each column were not statistically different at P < 0.05 (based on the LSD test).

## 4. Discussion

Limited research has been done on the reproductive biology of cassava after the publication of the protocol for controlled pollinations [9]. According to our knowledge, only Jos and co-workers have made additional contributions [13]. Cassava faces a peculiar situation. Being a key staple crop for millions of people (particularly in Sub-Saharan Africa) and an important commodity for industrial processes (particularly in SE Asia) it attracts today considerable interest from donors, investors and policymakers. Financial resources to support research in cassava have therefore increased significantly in recent years compared with the somber financial situation of the crop four decades ago [32]. However, as new technologies emerge, their application in cassava tends to be favored against that of more conventional or basic research. This situation is unfortunate because there are still large and relevant gaps of basic knowledge, which also affects the success of these new technologies being adapted to cassava. The first molecular map of cassava was published 20 years ago [33], and its genome has already been sequenced [34,35]. On the other hand, it is only recently that for the first time, the Mendelian segregation of a recessive trait was demonstrated in cassava thus confirming that it is indeed a functional diploid species [36]. Also recent is the first report on the induction of flowering [37]. The lack of knowledge regarding pollen tube growth and rate of embryo development has been particularly detrimental for the progress to develop a protocol for the production of doubled haploids in cassava [38].

The standard procedure used by many breeding programs relied on a 1980 publication [9], which states that cassava stigmas remain receptive for up to 24 h after anthesis. Covering the female flowers for just one DAP was therefore considered safe enough to prevent undesirable outcrosses and has been the standard procedure for decades. This procedure was suitable for the needs of different breeding programs when the objective was to generate genetic variability with a ‘*reasonable*’ certainty about the pedigree of each cross. However, the large increase in the research to develop molecular markers in the past two decades made it clear that the levels of outcrosses in mapping populations were unacceptably high. The need for basic information on the reproductive biology of cassava (even for critical issues like the duration in the receptivity of stigmas) highlights the peculiar situation this crop faces. Results provided in Figure 5 clearly demonstrated that stigmas remain receptive actually for up to three days after anthesis. Covering flowers for only one or two days has probably affected the pedigree of many populations used for QTL identification by allowing outcrosses that could not be detected on time. It has also affected the ongoing efforts to develop protocols for the production of doubled haploids leading to false positives. Stigmas normally drop from the pistil around four days after anthesis, as had happened in the last experiment in which only 3 of the 27 flowers collected 96 HAP had the stigmas still attached to the pistil (data not presented).

Results from Figure 5 are also useful to conclude that sexual reproduction of cassava is variable and relatively inefficient in agreement with earlier findings [13,39]. Even when pollinated flowers were not covered, only an average of 8% of pollinations resulted in fruit set (Figure 5(a)) and yielded just 5% of the expected number of seeds (Figure 5(b)). This figure also indicates that there is a large genetic variation in the reproductive capacity of different progenitors. Both plots illustrate what breeders have known for decades from field experience: some clones are good progenitors (regarding how easy it is for some clones to produce seeds in controlled pollinations), whereas others are remarkably ‘*shy*’ for seed production. The detailed information provided in Table 7 further illustrates the large variation in reproductive efficiency of different genotypes, particularly when used as female. One of the problems of controlled pollinations is that there is just one batch of pollen used in the process. In open pollinations, on the other hand, bees will keep visiting the flower several times, particularly during the anthesis day. Later the bracts of the flower tend to retract to the original position they held before anthesis, thus probably reducing the frequency of visits by bees. An additional factor in controlled pollinations and in this study may be the effect of covering inflorescences with the mesh bags. There may be some physical damage to the flowers and/or changes in temperature inside the bags affecting the growth of pollen tubes and the fertilization success rates.

Results from the second experiment reinforce the idea that stigmas remain receptive for at least three days because pollen tube growth could be observed in flowers pollinated three days after anthesis (Table 3). There is, however, a clear deterioration from the optimum results observed at anthesis day (in agreement with data from Figure 5). This experiment also provided the first insight (after the pioneering work by Jos and co-workers [13]) of the chronology of fertilization of cassava egg cells and the relatively slow growth of pollen tubes as it approaches the embryo sac.

The last three experiments provided interesting information regarding pollen tube growth through the pistil down towards the embryo sac. According to our observations, the obturator of cassava ovary does not seem to delay pollen tube growth as was found, for instance, in *Prunus persica* [40]. On the other hand, only 1–3 pollen tubes continue growing into the beak. Therefore, it is suggested here, that transition from the obturator to the beak is a pollen tube selection point in cassava. The subject needs further investigation, especially studies on embryo sac maturation before and during pollination. Distance wise, pollen tube growth is very fast the first few HAP, frequently reaching the nucellar beak within 6 h. The growth, however, slows down as PT approach the end of the nucellar beak (pseudomicropyle). PTT have been observed at the embryo sac as early as 26 HAP (Table 4). However, at each collection time and in different experiments, there is a sharp decrease between PTT in the pseudomicropyle and at the embryo sac (Table 4–7). Perhaps embryo sacs need to reach a physiological maturity before pollen tubes can access it and the presence of PT in the beak may promote such process. Pollen tubes could be observed reaching the embryo sac at 24 or 48 HAP in two different female cassava progenitors in an earlier report [13]. A delay in the arrival of pollen tubes into the embryo sac was also noticed suggesting that perhaps some sort of incompatibility (which did not prevent fertilization) may have taken place. These authors did not provide, however, a statistical assessment of pollen tube through time [13].

Results from the last two experiments are interesting. Pollen tubes grew considerably slowly compared with other evaluations. Perhaps this is result of the change of genotypes used in the different experiments. More likely, however, it was the difference in environmental conditions under which these experiments were conducted. Figure 4 provides records on rainfall 1980–2014 at CIAT’s Experimental Station as well as during the period in which these experiments were conducted. It is clear that all three experiments were conducted in conditions considerably drier than normal. It is also clear that rainfall was more abundant during Experiment 3 than in the last two experiments. Temperatures were not as extreme in Experiment 3 and the average was considerably lower (29.5°C) than in experiments 4 and 5 (33.7°C and 33.3°C, respectively). The maximum temperatures recorded during experiments 3, 4, and 5 were 31.1°C, 35.5°C and 34.3°C, respectively. The combination of these environmental conditions may explain the slower pollen tube growth in the fourth experiment. Nevertheless, this is just one of many possible explanations. This data, however, would support that environmental conditions indeed affect the efficiency of sexual reproduction of cassava. Data from Table 7 clearly reinforces that this efficiency is also genotype-dependent. There is very limited information regarding the genetic and environmental influence on cassava’s sexual reproductive biology.

As it can be observed, the number of flowers representing each genotype in Experiments 4 and 5 was variable (Table 1). This is the normal situation in cassava breeding because some materials have an erect plant architecture with late and scarce flowering, whereas other begin flowering soon after planting and they do so often and profusely. The latter ones are more likely to be used more frequently as progenitors. The samples of genotypes and number of flowers from each of them, therefore, are an adequate sampling of the variation to be expected in cassava breeding (e.g. crossing) activities.

There are key conclusions from this study that are relevant to breeders, molecular geneticist and cassava researchers. The information generated are particularly useful for the development of a protocol to produce doubled haploids in cassava through wide crosses or irradiated pollen.

- Stigmas remain receptive through at least three DAA. Therefore, flowers from directed crosses need to remain covered for at least three days (or until stigmas drop out) to protect them from outcrosses.
- There is wide genetic variation in the reproductive efficiency, particularly as a female progenitor.
- There is no evidence of differences in reproductive efficiency between self- and cross-pollinations.
- Growth of pollen tube is highly variable, even when analyzing the same female and male progenitors. Pollen tubes have been observed to reach the embryo sac as early as 26 HAP, but in most cases up to 96 hours (or more) are needed.
- Pollen tube growth is very fast during the first hours after pollination, reaching the pseudomicropyle 24 HAP at high frequency. However, tube growth slows down considerably thereafter, thus delaying the arrival at the embryo sac for 24–72 additional hours.
